# Real-World Impact of a BRCA Testing Protocol in Portugal

**DOI:** 10.7759/cureus.86732

**Published:** 2025-06-25

**Authors:** Mariana Malheiro, Bruno Silva, João Rosa, Tomás Costa

**Affiliations:** 1 Medical Oncology, Hospital CUF Tejo, Lisbon, PRT; 2 Oncology, Hospital de São Francisco Xavier, Lisbon, PRT; 3 Medical Oncology, Unidade Local de Saúde (ULS) Loures Odivelas, Loures, PRT; 4 Oncology, Unidade Local de Saúde (ULS) Amadora Sintra, Amadora, PRT; 5 Dermatology, Unidade Local de Saúde (ULS) Amadora Sintra, Amadora, PRT

**Keywords:** brca1/2, brca gene testing, breast cancer genetics, evidence-based medicine, genetic oncology

## Abstract

BRCA1 and BRCA2 are key tumor suppressor genes involved in DNA repair, and their mutations significantly increase the risk of breast, ovarian, prostate, and pancreatic cancers. Despite international guidelines recommending genetic testing in patients meeting specific criteria, BRCA testing remains underutilized in Portugal, limiting early diagnosis and personalized treatment opportunities. This multicenter quality-improvement study aimed to characterize baseline BRCA testing practices and assess the impact of implementing a standardized testing protocol across three oncology centers: Unidade Local de Saúde (ULS) Lisboa Ocidental, ULS Amadora Sintra, and ULS Loures Odivelas. The intervention included baseline data collection, development of a protocol based on international best practices, dissemination to clinical teams, and post-implementation evaluation. At baseline, the three institutions requested an average of 14.16 BRCA tests per month. Following protocol implementation, the average rose to 29.66 monthly tests, representing a 109.4% increase. All centers showed improved testing rates, with the most significant relative rise at ULS Amadora Sintra (388.8%). Although differences between centers likely reflect institutional variation in awareness and resources, these findings support the effectiveness of a protocol-driven approach to improving adherence to genetic testing guidelines. This study presents the first multicenter real-world data on BRCA testing practices in Portugal and underscores the value of local engagement and standardization in advancing precision oncology.

## Introduction

BRCA genes are classic tumor suppressor genes that maintain genomic stability by participating in DNA damage response (DDR) and DNA repair pathways [1], with mutations being associated with an increased risk of several types of cancer, including breast, ovarian, prostate, and pancreatic cancers [2-4].

Since the discovery of BRCA1 and BRCA2 [5,6], BRCA testing has been recognized as a valuable tool for the early diagnosis of these tumors, gaining further clinical relevance with the advent of PARP inhibitors, which have shown promising results in treating cancers harboring genetic defects in homologous recombination repair (HRR), particularly those involving BRCA mutations [7]. These findings highlight the need for testing guidelines aimed at identifying patients at increased risk for BRCA mutations, facilitating early diagnosis and enabling the personalization of treatment strategies based on genetic profiles.

However, despite the growing consensus and multiple testing recommendations [8], real-world data from Portugal on BRCA testing practices in healthcare institutions are limited, hindering efforts to assess compliance with testing guidelines and their real impact on patients’ lives. For instance, a 2023 study in pancreatic cancer patients [9] found that although 71% of oncologists recommended PARP inhibitors for those without disease progression after four months of chemotherapy, only about half routinely requested BRCA testing.

Given the paucity of information regarding BRCA testing patterns in Portuguese oncology centers, a multicenter study was designed involving the oncology services of Unidade Local de Saúde (ULS) Lisboa Ocidental, ULS Amadora Sintra, and ULS Loures Odivelas. These units were selected because they fall under the new organizational model of the Portuguese National Health Service-based on local health units (ULS) that integrate hospitals and primary care centers-thereby ensuring that the results obtained can be more readily replicated. As such, this study aimed to (1) quantify BRCA test utilization before and after protocol implementation and (2) assess whether a standardized institutional protocol improves testing adherence in routine clinical settings.

## Materials and methods

This was a multicenter study involving the oncology departments of ULS Lisboa Ocidental, ULS Amadora Sintra, and ULS Loures Odivelas. A baseline testing profile was gathered based on the number of BRCA test requests in each hospital.

A BRCA testing protocol was developed by the authors, based on international best practices (Table 1), detailing indications, the most frequent testing doubts, and the interpretation of post-testing results. The protocol was introduced during an oncology team meeting in each department. Clinicians were reminded of the importance of BRCA testing for optimizing patient care. A web portal was developed to allow clinicians to access testing indications in real time. This platform also housed supplementary oncology training resources-including instructional videos and a further 12 clinical action protocols-to encourage its regular use in clinical practice. Three months after the protocol was introduced, the number of test requests in each service was re-evaluated to measure the intervention’s impact.

**Table 1:** Clinical protocol for BRCA testing *In breast cancer patients whose test results could impact treatment decisions (e.g., systemic treatment with PARP inhibitors for metastatic disease or adjuvant olaparib therapy for high-risk, HER2-negative breast cancer), BRCA testing should also be considered. **BRCA testing can also be considered in patients with prostate cancer with intraductal, ductal, or cribriform histology; metastatic prostate cancer (hormone-sensitive or castration-resistant); high-risk prostate cancer (≥T3; Gleason score ≥ 8; prostate-specific antigen (PSA) ≥ 20) plus a family member diagnosed with prostate cancer at <60 years of age; prostate cancer with a family history of multiple cancers on the same side of the family; prostate cancer in a brother, father, or multiple family members (same side of the family) diagnosed with non-localized disease at <60 years of age or who died from prostate cancer.

Condition	Indications
Breast cancer*	Patients diagnosed at ≤50 years of age; patients with triple-negative breast cancer; patients with bilateral breast cancer, with at least one tumor diagnosed between 40 and 45 years of age (synchronous or metachronous); patients with lobular breast cancer and a personal or family history of diffuse gastric cancer; male patients with breast cancer at any age
Ovarian cancer	Patients with ovarian cancer at any age
Prostate cancer**	Metastatic castration-resistant prostate cancer, in patients eligible for PARP inhibitor therapy
Known family history	Family members who are carriers of a known BRCA1/2 mutation
Family history suggestive of BRCA mutation	≥1 first-degree relative with breast cancer at ≤50 years of age, or ovarian cancer, or pancreatic cancer, or metastatic/high-risk prostate cancer; ≥3 diagnoses of breast and/or prostate cancer (at any stage) on the same side of the family (including the patient)

Data were analyzed with SPSS, version 30.0 (IBM Corp., Armonk, NY, US, 2025). Descriptive statistics were presented as means and standard deviations after checking for normality assumptions, namely, the Shapiro-Wilk test, suited for samples lower than 50 and kurtosis and asymmetry coefficients lower than 2 or higher than -2 [10]. Baseline and average three-month comparisons were performed with paired sample t-tests. Effect size was calculated as Cohen’s d, following Cohen’s [11] recommendations for 0.02 (low), 0.05 (medium), and 0.08 (high). Significance was deemed for p < 0.10, acknowledging the low sample size [12,13].

## Results

**Figure 1:**
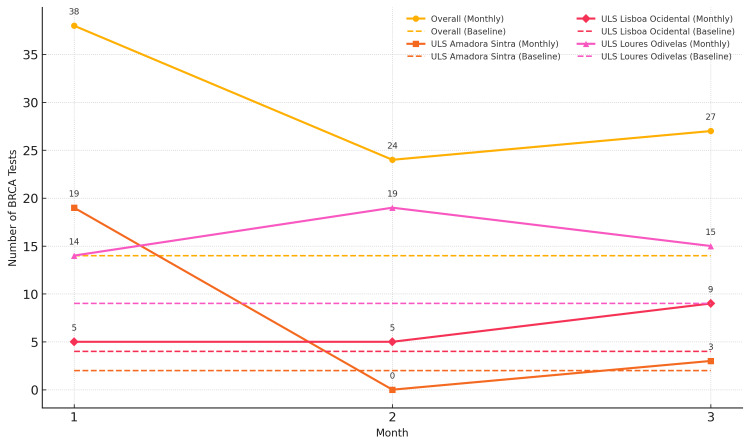
BRCA testing profile ULS: Unidade Local de Saúde

BRCA testing requests were analyzed in the three selected centers over the six months preceding the protocol presentation meeting at each unit (baseline). Table 2 and Figure 1 show descriptive results for the number of BRCA test requests.

**Table 2:** Number of BRCA test requests *Baseline average was determined from the mean number of tests performed during the six months preceding the protocol presentation. **Post-protocol average was calculated from the mean number of tests conducted in the three months that followed the protocol presentation. ULS: Unidade Local de Saúde

Institution	BRCA tests				
	Baseline average*	Month 1	Month 2	Month 3	Post-protocol average**
ULS Lisboa Ocidental	4.0	5	5	9	6.3
ULS Amadora Sintra	1.5	19	0	3	7.3
ULS Loures Odivelas	8.7	14	19	15	16
Overall	14.2	38	24	27	29.7

During the baseline period, the three institutions together performed an average of 14.2 BRCA tests per month. Individually, ULS Loures Odivelas contributed the largest share at baseline (8.7 tests), followed by ULS Lisboa Ocidental (4.0 tests) and ULS Amadora Sintra (1.5 tests).

In Month 1, the total number of tests rose to 38. ULS Amadora Sintra drove this increase, jumping from 1.5 at baseline to 19 tests (an increase of 17.5 tests), while ULS Loures Odivelas increased from 8.7 to 14 and ULS Lisboa Ocidental registered a slight increase, from 4.0 to 5.

During Month 2, overall testing fell to 24. ULS Loures Odivelas maintained high activity with 19 tests, whereas ULS Lisboa Ocidental remained stable at five tests and ULS Amadora Sintra dropped to zero.

In Month 3, the combined total was 27 tests: ULS Loures Odivelas performed 15, ULS Lisboa Ocidental nine, and ULS Amadora Sintra recovered partially with three tests.

Across Months 1-3, the average monthly volume reached 29.7 tests. Institution-level three-month means were 16.0 tests at ULS Loures Odivelas, 7.3 at ULS Amadora Sintra, and 6.3 at ULS Lisboa Ocidental. Thus, while ULS Loures Odivelas consistently led testing activity, ULS Amadora Sintra exhibited the greatest month-to-month volatility, with a pronounced peak in Month 1 and a trough in Month 2.

## Discussion

In patients carrying a BRCA1 mutation, the average cumulative risk of developing breast cancer by the age of 70 is approximately 65%, whereas the risk for ovarian cancer is around 39% [14]. Despite this elevated risk, some studies estimate that fewer than 30% of individuals who meet screening criteria actually undergo testing [15,16], and certain authors highlight that even these criteria may exclude a significant number of eligible patients [17].

Although this undertesting can partly be attributed to structural or testing capacity constraints [18], the lack of real-world data on the number of BRCA tests performed at each center hinders the identification of testing barriers and the development of targeted strategies to optimize clinical practice. Nevertheless, in the limited number of cases described where standardized testing protocols have been successfully implemented and healthcare professionals engaged at the local level, adherence to best practices in BRCA testing has risen dramatically [19].

Our multicenter study corroborates these observations by demonstrating that a protocol-driven approach can substantially increase BRCA testing rates in Portuguese oncology centers. Following the implementation of a standardized protocol, the monthly average of requested tests increased from 14.16 to 29.66, representing a 109.4% relative rise. These findings are in line with international data; for instance, Mendenhall et al. [20], in a similar study, implemented a streamlined genetic screening workflow and reported a 10-fold increase in testing over two years. Their study proposed a scalable model for replication in other centers, and our data reinforce the feasibility of such an approach by providing the first multicenter real-world evidence on BRCA testing optimization.

Although all centers showed a significant rise in the number of tests, there was notable variability among the three participating institutions. Differences in institutional resources, patient populations, and awareness of genetic testing are likely contributors. For example, ULS Amadora Sintra demonstrated the most pronounced increase (388.8%), possibly reflecting previously underutilized testing capacity or a rapid shift in clinical practice driven by heightened awareness of BRCA mutational status.

Despite these encouraging results, certain limitations warrant caution. First, the three-month follow-up period may be insufficient to evaluate the long-term sustainability of these findings; this limitation could be mitigated by ongoing result-presentation sessions, in which each service’s actual versus expected test volumes are reported to enable continuous performance monitoring. Second, because the study was designed as a quality-improvement intervention rather than a formal hypothesis-driven trial, it does not allow for robust statistical inference regarding causality. Confounding variables-such as evolving clinical guidelines, new data supporting broader indications for BRCA testing, and local policy changes-could also have influenced the observed testing rates. Furthermore, only absolute test numbers were tracked, without measuring the expected number of tests (i.e., patients meeting testing criteria out of the total number of patients seen at each center).

Nevertheless, this study remains important as one of the few published investigations presenting real-world data on BRCA testing in Portugal, since previous Portuguese studies have generally featured retrospective analyses of specific patient cohorts to identify testing criteria [21] or were designed to improve follow-up in high-risk populations [22]. Future studies incorporating long-term follow-up data on patient outcomes (e.g., impact on therapeutic decision-making and survival) will be essential to determine the benefits of broadly implementing these programs in other institutions.

## Conclusions

While this study’s limited follow-up duration, sample size, and data granularity temper its generalizability, our results nonetheless suggest that a standardized, context-tailored BRCA testing protocol can narrow the gap between guideline recommendations and real-world practice. To secure and reproduce these gains, future efforts should include larger, multicenter cohorts, extended monitoring periods, ongoing educational initiatives, and real-time performance feedback-steps that will ultimately support more personalized, evidence-based oncological care in Portugal and beyond.
